# Response of the goat mammary gland to infection with *Staphylococcus aureus* revealed by gene expression profiling in milk somatic and white blood cells

**DOI:** 10.1186/1471-2164-13-540

**Published:** 2012-10-09

**Authors:** Paola Cremonesi, Rossana Capoferri, Giuliano Pisoni, Marcello Del Corvo, Francesco Strozzi, Rachel Rupp, Hugues Caillat, Paola Modesto, Paolo Moroni, John L Williams, Bianca Castiglioni, Alessandra Stella

**Affiliations:** 1 Istituto di Biologia e Biotecnologia Agraria, Consiglio Nazionale delle Ricerche, via Einstein, Lodi, 26900, Italy; 2 IDRA-LAB Istituto Sperimentale Italiano “L. Spallanzani”, via Einstein, Lodi, 26900, Italy; 3 Dipartimento di Scienze Veterinarie per la Salute, la Produzione Animale e la Sicurezza Alimentare, Università degli Studi di Milano, via Celoria 10, Milano, 20133, Italy; 4 Parco Tecnologico Padano, via Einstein, Lodi, 26900, Italy; 5 INRA, UR631, Station d’Amélioration Génétique des Animaux, Castanet-Tolosan, F-31326, France; 6 Istituto Zooprofilattico Sperimentale del Piemonte, Liguria e Valle d’Aosta, via Bologna 148, Torino, Italy; 7 Quality Milk Production Services, Cornell University, 240 Farrier Road, Ithaca, NY, 14853, USA

## Abstract

**Background:**

*S. aureus* is one of the main pathogens responsible for the intra-mammary infection in dairy ruminants. Although much work has been carried out to understand the complex physiological and cellular events that occur in the mammary gland in response to *S. aureus*, the protective mechanisms are still poorly understood. The objectives of the present study were to investigate gene expression during the early response of the goat mammary gland to an experimental challenge with *S. aureus,* in order to better understand the local and systemic response and to compare them in two divergent lines of goat selected for high and low milk somatic cell scores.

**Results:**

No differences in gene expression were found between high and low SCS (Somatic Cells Score) selection lines. Analysing the two groups together, an expression of 300 genes were found to change from T0 before infection, and T4 at 24 hours and T5 at 30 hours following challenge. In blood derived white blood cells 8 genes showed increased expression between T0 and T5 and 1 gene has reduced expression. The genes showing the greatest increase in expression following challenge (5.65 to 3.16 fold change) play an important role in (i) immune and inflammatory response (*NFKB1*, *TNFAIP6*, *BASP1*, *IRF1*, *PLEK*, *BATF3*); (ii) the regulation of innate resistance to pathogens (*PTX3*); and (iii) the regulation of cell metabolism (*CYTH4*, *SLC2A6*, *ARG2*). The genes with reduced expression (−1.5 to −2.5 fold) included genes involved in (i) lipid metabolism (*ABCG2*, *FASN*), (ii) chemokine, cytokine and intracellular signalling (*SPPI*), and (iii) cell cytoskeleton and extracellular matrix (*KRT19*).

**Conclusions:**

Analysis of genes with differential expression following infection showed an inverse relationship between immune response and lipid metabolism in the early response of the mammary gland to the *S. aureus* challenge. *PTX3* showed a large change in expression in both milk and blood, and is therefore a candidate for further studies on immune response associated with mastitis.

## Background

Mastitis is an inflammation of the mammary gland to infection, and is usually caused by bacteria. Mastitis represents one of the most economically important health traits for milk production, which makes it among the major concerns for the livestock sector [1]. Although much work has been carried out in dairy ruminants to understand the complex physiological and cellular events that occur in the mammary gland in response to pathogens [2-4], the protective mechanisms are still poorly understood. Invading pathogens activate the immune defence in the udder, which is a complex biological process involving not only resident and recruited immune cells, but also mammary epithelial and endothelial cells. The result is an increase in the number of somatic cells in milk. Polymorphic nuclear neutrophil granulocytes are the predominant cell type recruited to the gland [5,6], and their numbers increase 10- to 50-fold during the first few hours following infection [7,8]. The aetiology of the pathogens influences the severity of the symptoms: contagious pathogens, such as *Staphylococcus aureus* or *Streptococcus agalactiae*, cause ongoing chronic disease and sub-clinical mastitis, while the environmental coliform bacteria often cause acute, clinical mastitis [9]. Variation in presentation of the disease between pathogens may be the result of differences in the capability of the innate immune system to mount initial defences. This may be linked to factors such as recognition of pathogen derived antigen e.g. by Toll-like receptors (TLR) or the mobilisation of bactericidal effector molecules such as the β-defensins.

Immediate and appropriate recognition of the invading pathogen is fundamental for the prompt and proper activation of the immune response of the host. Infection will only be able to develop and the disease become manifest if these mechanisms fail. Studies in humans and model organisms have revealed that receptors and effector molecules of the innate immune system are a crucial first line of disease defence. An infection sets in motion a, normally, well ordered cascade of defence mechanisms to eliminate the pathogen, in which innate and adaptive immune mechanisms cooperate. However, there are no consistent descriptions of mastitis-related mammary gland-specific expression of the key factors controlling the innate immune system. Previous analyses have been focused on effector mechanisms in later stages of the adaptive immune response to an infection [10], studies of the early phases of infection which could shed light on innate defence mechanisms process in the udder are currently missing. Such studies can only be achieved in controlled infection experiments where the time of infection and pathogen involved are known.

Disease response is a complex trait under multi-genic control, which makes it difficult to develop appropriate genetic selection strategies for improved immune response. Nevertheless, a genetic component of host responses to bacteria during intra-mammary infections has been widely documented, and mastitis has a heritability up to 20% in goats [11,12]. Breeding to improve resistance to mastitis has had limited success, as very little is known about the genetic basis of resistance and functional complexity of the host pathogen interaction during infection. The overriding problems of developing an appropriate selection strategy to control mastitis are (i) the plethora of pathogens that cause mastitis, each of which may elicit a different immune response, and (ii) the strong environmental and management effects on the incidence of mastitis [13]. The number of somatic cells in milk is correlated with intra-mammary infection and cattle breeders have used somatic cell scores (SCS) in genetic selection for reducing mastitis. However, it is not certain how well SCS predicts immune response to infection with a mastitis causing pathogens, or incidence of clinical mastitis. Nevertheless, breeding programmes for mastitis resistance in dairy cattle [14,15] and sheep [16] have used SCS as a selection criterion. For goats, the relevance of SCS as a predictor of parameters of udder health and the susceptibility against mastitis is still untested.

Over the last decade techniques for studying the transcriptome have improved dramatically, in particular the use of microarrays that represent a large proportion of the expressed genes. The application of these approaches for dairy ruminants transcriptome profiling has identified genes, pathways and regulatory networks activated in mammary tissues during experimental infection by various pathogens, including *E. coli*, *S. aureus* and *S. uberis*[10,17-21]. The meta-analysis of transcription-profiling data from six independent studies of the mammary gland infected with different pathogens identified common signatures of infection among species and that were characteristic of early vs late stage responses [22].

The objectives of the present study were to investigate the early response of goats to a controlled infection with *S. aureus* by following gene expression responses in immune related cells in the blood and milk in order to better understand the local and systemic response. The study also compared the responses of two divergent lines of goat selected for high and low milk SCS.

## Results

### Intra-mammary *S. aureus* growth following experimental infection

**Figure 1:**
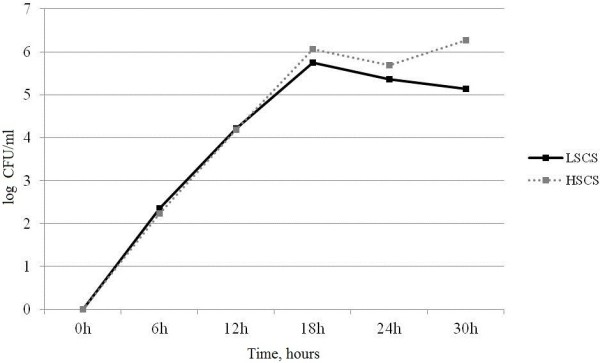
***S. aureus *counts in milk from LSCS (Low Somatic Cell Score) and HSCS (High Somatic Cell Score) goats at different time points.** Mean *S. aureus* log10 CFU/ml in milk was measured in LSCS and HSCS goats at 0, 6, 12, 18, 24 and 30 hours post infection. *S. aureus* counts peaked at 18 h post-challenge with means of 5.8 log10 CFU/ml in LSCS line animals and 6.1 log10 CFU/ml HSCS selection line goats. From 18 h to 30 h this value remained constant (paired t-test).

Following intra-mammary experimental infection with *S. aureus,* the pathogen could be cultured from the milk of all 10 experimentally infected animals at 6 hours, and at the final sampling 30h post-infection, *S. aureus* could be cultured from 9 of the 10 infected animals. *S. aureus* present in milk samples reached a maximum at 18h post-infection with a mean of 5.8 log10 CFU/ml (Colony Forming Unit/ml) in the Low SCS (LSCS) line animals and 6.1 log10 CFU/ml the High SCS (HSCS) selection line goats. The number of *S. aureus* then remained constant for the rest of the study period (Figure 1). Strain analysis using the RAPD-PCR (Randomly Amplified Polymorphic DNA-PCR) method confirmed that *S. aureus* isolated from infected udders were the same as the strain used for the infection (data not shown). The control PBS-infused udders remained free from detectable infection throughout the study. No significant difference was observed in *S. aureus* counts at different time points in milk from LSCS and HSCS goats.

### Systemic and localized inflammatory responses to *S. aureus* intra-mammary infection

**Figure 2:**
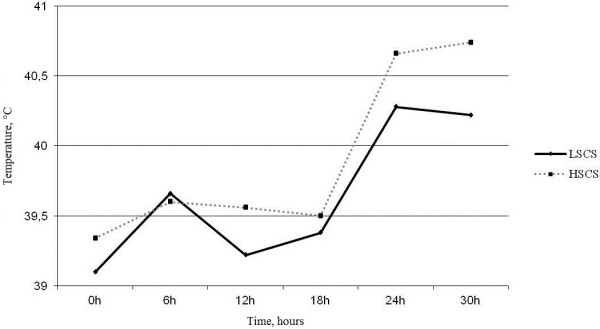
**Mean body temperature of LSCS (Low Somatic Cell Score) and HSCS (High Somatic Cell Score) goats.** Rectal temperature was monitored throughout the study. A temperature increase was only significant (paired t-test, p=0.05) at the 24 and 30 h time points and reached a maximum 30 h after infection. A paired t-test was applied to milk SCC of right udders in LSCS and HSCS goats to test if the changes at each time point were significant. No significant difference was observed in mean body temperature of LSCS and HSCS goats at different time points.

As an indicator of a systemic response to *S. aureus* infection, rectal temperatures were monitored throughout the study. Body temperature showed an upward trend from 0 to 18 h post-infection, however, the increased temperature was only significant (p=0.05) at the 24 and 30 h time points. Temperatures reached a maximum 30 h after infection and reached a peak mean (± S.D.) of 40.2 (±0.5) and 40.7 (±0.6) °C in LSCS and HSCS goats, respectively, which is not a significant difference (Figure 2).

**Figure 3:**
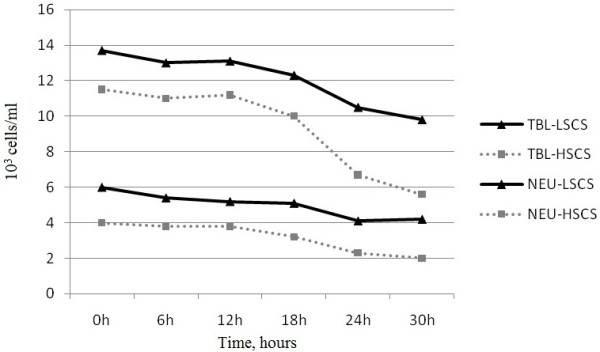
**Total blood leukocyte (TBL) and of neutrophils (NEU) counts of LSCS (Low Somatic Cell Score) and HSCS (High Somatic Cell Score) goats.** 18 h after challenge the TBL and NEU counts were 13,7 and 6 x 10^3^ cells/ml in LSCS goats and 11,5 and 4 x 10^3^ cells/ml in HSCS goats, respectively. The lowest counts were reached at 30h post-infection (paired t-test).

The systemic response to intra-mammary infection with *S. aureus* was characterized by a significant decrease (p=0.05) of total blood leukocyte (TBL) and of neutrophil (NEU) numbers 18h post-infection. The TBL and NEU counts were 13.7 and 6.0 x 10^3^ cells/ml in LSCS goats and 11.5 and 4.0 x 10^3^ cells/ml in HSCS goats. The lowest blood cell counts were reached at 30h post-infection, when the TBL and NEU counts were 9.8 and 4.2 x 10^3^ and 5.6 and 2.0 x 10^3^ cells/ml in LSCS and HSCS goats, respectively (Figure 3).

**Figure 4:**
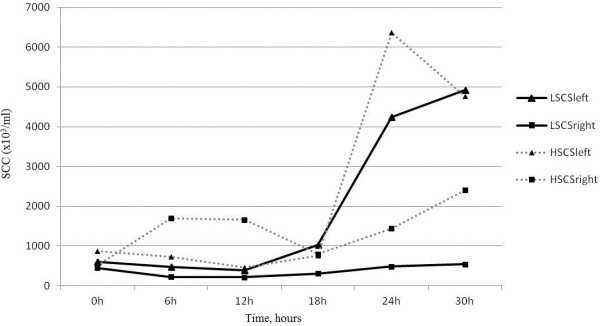
**Somatic cell count of LSCS (Low Somatic Cell Score) and HSCS (High Somatic Cell Score) goats in right and left udders.** Mean milk SCC (x10^3^/ml) of LSCS and HSCS goats in left udders (*S. aureus*-infected) and in right udders (PBS-infused) was measured at 0, 6, 12, 18, 24 and 30 hours post infection. Increases in milk SCC were initially observed after 18 h of infection. Milk SCC in the right hand control udders remained unchanged throughout the study in LSCS goats, while milk SCC in left hand udder halves of HSCS goats increased twofold (paired t-test, p=0.05).

Changes in milk SCC (Somatic Cell Count) were monitored as an indicator of local response. Mean milk SCCs (x10^3^/ml), before *S. aureus* intra-mammary infection, were 6.0 x10^5^ and 4.5x10^5^ for LSCS left and right udder halves respectively, and 8.8 x10^5^ and 5.2 x10^5^ for HSCS left and right udder halves respectively at T5. Increases in milk SCC were initially observed after 18 h of infection. Milk SCC in the right hand control udders, infused with saline, remained unchanged throughout the study in LSCS goats, while milk SCC in right hand udder halves of HSCS goats increased significantly (p=0.05) (Figure 4).

The differential milk somatic cell composition was compared between LSCS and HSCS goats before and following infection. The mean proportion of macrophages in LSCS (19.98%) and HSCS (11.39%) prior to challenge was statistically significant (p=0.030; Table 1). After the challenge, for LSCS a significant increase in neutrophils was observed at T4 vs T0 (p=0.0031) and T5 vs T0 (p=0.0010) and decrease was observed for macrophages (T4 vs T0, p=0.0085; T5 vs T0 p=0.0009), lymphocytes (T4 vs T0, p=0.014; T5 vs T0, p=0.016) and epithelial cells (T4 vs T0, p=0.014; T5 vs T0, p=0.049) (Additional file 1). For HSCS a significant increase in neutrophils was found at T5 vs T0 (p=0.028), while significant decreases were seen for macrophages (T4 vs T0, p=0.016; T5 vs T0, p=0.010) and for epithelial cells (T5 vs T0, p=0.027).

**Table 1:** Differential cell counts (DCCs) in milk somatic cells in LSCS and HSCS animals during five time points post-infection. Mean and Standard Deviation (Dev. St) of the percentages of polymorfonuclear (PMN), macrophages (M), lymphocytes (L) and epithelial cells (E) at all time points are indicated

		**Mean T0**	**Dev. St**	**Mean T1**	**Dev. St**	**Mean T2**	**Dev. St**	**Mean T3**	**Dev. St**	**Mean T4**	**Dev. St**	**Mean T5**	**Dev. St**
LSCS	PMN	52.43	11.16	55.14	10.70	58.26	12.72	65.80**	10.05	83.55***	4.99	85.21***	7.78
	M	19.98	6.34	12.89	2.92	14.57	8.32	10.88**	4.93	4.76***	3.08	4.41***	4.92
	L	8.26	4.37	10.35	5.73	9.11	5.32	5.35	2.46	1.49**	1.07	0.20**	0.45
	E	19.32	3.47	21.62	9.53	18.06	7.58	17.98	7.20	10.20**	3.68	10.18**	4.68
HSCS	PMN	56.26	17.11	58.41	12.09	55.05	8.57	69.33	19.46	82.64	8.36	84.50**	8.58
	M	11.39	2.02	11.72	2.55	8.62**	2.84	5.30**	2.73	4.09**	2.58	4.61**	2.83
	L	9.15	9.24	9.80	4.16	7.78	7.83	1.97	2.08	0.91	1.55	0.55	0.80
	E	23.21	8.42	20.07	7.02	28.55	9.20	23.41	20.12	12.36	6.83	10.35**	6.33

**p-value < 0.05; ***p-value < 0.01.

Therefore, statistically different increase and variations in somatic cell composition were seen in the in right hand udder halves of HSCS vs LSCS goats (Figure 4 and Table 1), whereas there were no statistically significant differences in either blood or milk between the HSCS and LSCS goats for the other phenotypic responses (*i.e.* rectal temperature, milk SCC, TBL, NEU) at any of the time points after infection.

### Gene expression analysis of milk samples

No differences in gene expression were found at any of the time points between the two selection lines (HSCS vs LSCS). Analysing the two groups together (HSCS + LSCS), a total of 300 genes were found to be differentially expressed (p value < 0.01 and log2 fold change > 1.5) in milk SCC between T0 and T4 and 128 genes between T0 and T5. Among these genes, the majority (251 for T4 vs T0 and 123 for T5 vs T0) showed an increase in expression. Ingenuity Pathway Analysis (IPA; Ingenuity Systems, Inc.), was able to identify 259 and 127 genes respectively for T4 and T5 of milk samples, based on comparative annotation using human or mouse orthologs within the IPA Knowledge base (Additional files 2 and Additional file 3). The 10 genes in milk somatic cells (MSC) showing the greatest increase in expression post infection between 5.6 and 3.2 fold (Table 2) play an important role in (i) immune and inflammatory response (*NFKB1*, *TNFAIP6*, *BASP1*, *IRF1*, *PLEK*, *BATF3*); (ii) the regulation of innate resistance to pathogens (*PTX3*); and (iii) the regulation of cell metabolism (*CYTH4*, *SLC2A6*, *ARG2*). Fewer genes showing reduced expression between −1.5 to −2.5 fold (Table 3) were involved in (i) lipid metabolism (*ABCG2*, *FASN*), (ii) chemokine, cytokine and intracellular signalling (*SPPI*), and (iii) the cytoskeleton and extracellular matrix (*KRT19*).

**Table 2:** **List of the top 10 genes with the greatest increase in expression post infection in milk somatic cells due to intra-mammary infection with *S. aureus***

**Gene symbol**	**Gene name**	**Primary functions**	**Log fold change**
T4			
*PTX3*	Pentraxin 3, long	regulation of innate resistance to pathogens, inflammatory reactions, possibly clearance of self-components and female fertility	5.6
*PLEK*	Pleckstrin	Major protein kinase C substrate of platelets	4.4
*IRF1*	interferon regulatory factor 1	binds to the upstream regulatory region of type I IFN and IFN-inducible MHC class I genes (the interferon consensus sequence (ICS)) and activates those genes. Acts as a tumor suppressor	4.3
*NCF1*	neutrophil cytosolic factor 1	activation of the latent NADPH oxidase (necessary for superoxide production)	4.0
*SLC2A6*	solute carrier family 2 (facilitated glucose transporter), member 6	Facilitative glucose transporter; binds cytochalasin B with low affinity	3.9
*BASP1*	brain abundant, membrane attached signal protein 1	encodes a membrane bound protein with several transient phosphorylation sites and PEST motifs	3.9
*CYTH4*	cytohesin 4	Promotes guanine-nucleotide exchange on ARF1 and ARF5. Promotes the activation of ARF through replacement of GDP with GTP	3.9
*TNFAIP6*	tumor necrosis factor, alpha-induced protein 6	involved in cell-cell and cell-matrix interactions during inflammation and tumorigenesis	3.8
*COL3A1*	collagen, type III, alpha 1	Collagen type III occurs in most soft connective tissues along with type I collagen	3.8
*BATF3*	basic leucine zipper transcription factor, ATF-like 3	negative regulator of AP-1-mediated transcription by heterodimerizing with JUN and binding DNA at 12-O-tetradecanoylphorbol-13-acetate response elements (TRE) (consensus: 5′-TGA[CG]TCA-3′). Represses IL2 and MMP1 promoter activities	3.7
T5			
*PTX3*	Pentraxin 3, long	regulation of innate resistance to pathogens, inflammatory reactions, possibly clearance of self-components and female fertility	5.3
*S100A9*	S100 calcium binding protein A9	Calcium-binding protein. Has antimicrobial activity towards bacteria and fungi. Important for resistance to invasion by pathogenic bacteria. Up-regulates transcription of genes that are under the control of NF-kappa-B. Plays a role in the development of endotoxic shock in response to bacterial lipopolysaccharide (LPS) (By similarity). Promotes tubulin polymerization when unphosphorylated. Promotes phagocyte migration and infiltration of granulocytes at sites of wounding. Plays a role as a pro-inflammatory mediator in acute and chronic inflammation and up-regulates the release of IL8 and cell-surface expression of ICAM1. Extracellular calprotectin binds to target cells and promotes apoptosis. Antimicrobial and proapoptotic activity is inhibited by zinc ions	3.8
*ICAM1*	intercellular adhesion molecule 1	During leukocyte trans-endothelial migration, ICAM1 engagement promotes the assembly of endothelial apical cups through ARHGEF26/SGEF and RHOG activation. In case of rhinovirus infection acts as a cellular receptor for the virus	3.7
*SOD2*	superoxide dismutase 2, mitochondrial	Destroys radicals which are normally produced within the cells and which are toxic to biological systems	3.5
*PLEK*	Pleckstrin	Major protein kinase C substrate of platelets	3.4
*S100A8*	S100 calcium binding protein A8	Calcium-binding protein. Has antimicrobial activity towards bacteria and fungi. Important for resistance to invasion by pathogenic bacteria. Up-regulates transcription of genes that are under the control of NF-kappa-B. Plays a role in the development of endotoxic shock in response to bacterial lipopolysaccharide (LPS) (By similarity). Promotes tubulin polymerization. Promotes phagocyte migration and infiltration of granulocytes at sites of wounding. Plays a role as pro-inflammatory mediator in acute and chronic inflammation and up-regulates the release of IL8 and cell-surface expression of ICAM1. Extracellular calprotectin binds to target cells and promotes apoptosis. Antimicrobial and proapoptotic activity is inhibited by zinc ions	3.4
*DNAJB6*	DnaJ (Hsp40) homolog, subfamily B, member 6	Plays an indispensable role in the organization of KRT8/KRT18 filaments. Acts as an endogenous molecular chaperone for neuronal proteins including huntingtin. Has a stimulatory effect on the ATPase activity of HSP70 in a dose-dependent and time-dependent manner and hence acts as a co-chaperone of HSP70. Reduces huntingtin aggregation associated with HD. Also reduces cellular toxicity and caspase-3 activity	3.2
*TNFAIP6*	tumor necrosis factor, alpha-induced protein 6	involved in cell-cell and cell-matrix interactions during inflammation and tumorigenesis	3.1
*NCF4*	neutrophil cytosolic factor 4, 40kDa	Component of the NADPH-oxidase, a multicomponent enzyme system responsible for the oxidative burst in which electrons are transported from NADPH to molecular oxygen, generating reactive oxidant intermediates. It may be important for the assembly and/or activation of the NADPH-oxidase complex	3.1
*STEAP4*	STEAP family member 4	Metalloreductase that has the ability to reduce both Fe(3+) to Fe(2+) and Cu(2+) to Cu(1+). Uses NAD(+) as acceptor. Play a role in systemic metabolic homeostasis, integrating inflammatory and metabolic responses (By similarity). Associated with obesity and insulin-resistance. Involved in inflammatory arthritis, through the regulation of inflammatory cytokines. Inhibits anchorage-independent cell proliferation	3.1

**Table 3:** **List of the top 10 genes with an expression decrease in milk somatic cells due to intra-mammary infection with *S. aureus***

**Gene symbol**	**Gene name**	**Primary functions**	**Log fold change**
T4			
*SPP1*	secreted phosphoprotein 1	Acts as a cytokine involved in enhancing production of interferon-gamma and interleukin-12 and reducing production of interleukin-10 and is essential in the pathway that leads to type I immunity (By similarity)	−2.4
*ABCG2*	ATP-binding cassette, sub-family G (WHITE), member 2	Xenobiotic transporter that may play an important role in the exclusion of xenobiotics from the brain. May be involved in brain-to-blood efflux. Appears to play a major role in the multidrug resistance phenotype of several cancer cell lines. When overexpressed, the transfected cells become resistant to mitoxantrone, daunorubicin and doxorubicin, display diminished intracellular accumulation of daunorubicin, and manifest an ATP-dependent increase in the efflux of rhodamine 123	−2.1
*CD24*	CD24 molecule	Modulates B-cell activation responses. Signaling could be triggered by the binding of a lectin-like ligand to the CD24 carbohydrates, and transduced by the release of second messengers derived from the GPI-anchor. Promotes AG-dependent proliferation of B-cells, and prevents their terminal differentiation into antibody-forming cells	−2.0
*KRT19*	keratin 19	Involved in the organization of myofibers. Together with KRT8, helps to link the contractile apparatus to dystrophin at the costameres of striated muscle	−2.0
*EXOSC2*	exosome component 2	Component of the exosome 3′->5′ exoribonuclease complex, a complex that degrades inherently unstable mRNAs containing AU-rich elements (AREs) within their 3′ untranslated regions. Required for the 3′processing of the 7S pre-RNA to the mature 5.8S rRNA. Has a 3′-5′ exonuclease activity	−1.9
*HDAC10*	histone deacetylase 10	Responsible for the deacetylation of lysine residues on the N-terminal part of the core histones (H2A, H2B, H3 and H4). Histone deacetylation gives a tag for epigenetic repression and plays an important role in transcriptional regulation, cell cycle progression and developmental events. Histone deacetylases act via the formation of large multiprotein complexes	−1.9
*C5ORF56*	chromosome 5 open reading frame 56	Homo sapiens chromosome 5 open reading frame 56 (C5orf56), mRNA	−1.9
*ELF5*	E74-like factor 5 (ets domain transcription factor)	Transcriptionally activator that may play a role in regulating the later stages of keratinocytes terminal differentiation	−1.8
*MID2*	midline 2	The protein encoded by this gene is a member of the tripartite motif (TRIM) family. The TRIM motif includes three zinc-binding domains, a RING, a B-box type 1 and a B-box type 2, and a coiled-coil region. The protein localizes to microtubular structures in the cytoplasm.	−1.8
*REG3G*	RNA binding motif protein 5	Might be a stress protein involved in the control of bacterial proliferation (By similarity)	−1.8
T5			
*FASN*	fatty acid synthase	Fatty acid synthetase catalyzes the formation of long-chain fatty acids from acetyl-CoA, malonyl-CoA and NADPH. This multifunctional protein has 7 catalytic activities and an acyl carrier protein	−2.1
*CCNB2*	cyclin B2	Essential for the control of the cell cycle at the G2/M (mitosis) transition	−1.5

### Canonical pathway

The most represented canonical signalling and metabolic pathways among the differentially expressed genes at T4 and T5 included *MIF-mediated Glucocorticoid Regulation*, *MIF Regulation of Innate Immunity*, *NF-kB Signalling*, *IL-10 Signalling* and *Hypoxia Signalling in Cardiovascular System* for T4 and *Production of Nitric Oxide and Reactive Oxygen Species in Macrophages*, LXR/RXR *Activation, Toll-like Receptor Signalling*, *Acute Phase Response Signalling* and *MIF-mediated Glucocorticoid Regulation* for T5 (Table 4 and Additional file 4). The majority of genes with > 1.5 fold change in expression between T0 and T5 within these pathways had an increase in expression. Among the five pathways, three were related to immune or inflammatory functions: *IL-10 Signalling*, which limits the inflammatory response, *Production of Nitric Oxide and Reactive Oxygen Species in Macrophages* and *MIF-mediated Glucocorticoid regulation*, which promote the inflammatory response. The pathway analysis also revealed that the *Toll-like Receptor Signalling* pathway was activated. Toll-like receptor activation is known to stimulate the synthesis of pro-inflammatory cytokines and chemokines in response to the bacterial infection, and the *LXR/RXR Activation pathway* which is involved in inflammation and lipid metabolism.

**Table 4:** Most significant affected IPA canonical pathways

**Canonical pathway (IPA)**	**Genes**	**P-value**	**Ratio**
T4			
MIF-mediated Glucocorticoid Regulation	*TLR4, NFKBIA, CD14, PTGS2, NFKBIB, NFKB1*	4.67E-06	6/41
MIF Regulation of Innate Immunity	*TLR4, NFKBIA, CD14, PTGS2, NFKBIB, NFKB1*	1.8E-05	6/49
NF-κB Signalling	*TLR4, IL1A, TGFBR1, RIPK1, NFKBIA, MYD88, BMP2, RELB, TNFAIP3, NFKBIB, NFKB1*	2.46E-05	11/176
IL-10 Signalling	*IL18RAP, IL1A, NFKBIA, CD14, ARG2, NFKBIB, NFKB1*	4.47E-05	7/78
Hypoxia Signalling in the Cardiovascular System	*HSP90B1, UBE2H (includes EG:7328), NFKBIA, UBE2B, HIF1A, NFKBIB, UBE2L6*	5.4E-05	7/71
T5			
Production of Nitric Oxide and Reactive Oxygen Species in Macrophages	*TLR4, PPP1R3D, NFKBIA, ARG2, NCF4, NFKB1, RHOH, IRF1, SIRPA*	1.98E-06	9/189
LXR/RXR Activation	*TLR4, IL18RAP, FASN, CD14, ARG2, NFKB1*	1.85E-05	6/93
Toll-like Receptor Signalling	*TLR4, NFKBIA, MYD88, CD14, NFKB1*	2.72E-05	5/55
Acute Phase Response Signalling	*SOD2, C3, NFKBIA, MYD88, OSM, SERPINA1, NFKB1, SAA1*	3.59E-05	8/183
MIF-mediated Glucocorticoid Regulation	*TLR4, NFKBIA, CD14, NFKB1*	8.09E-05	4/41

Five most significant canonical pathways identified with IPA using the significantly affected genes for T_4_ and T_5_. The identified canonical pathways are listed from the lowest to the highest p-value, and are reported with the involved genes and the corresponding ratio (# of genes involved/ # of known genes in the pathway).

At T4 and T5, 19 and 11 networks were identified using Ingenuity pathway analysis, respectively. The T4 networks contained a total of 244 differentially expressed genes which were involved in pathways and functions including the *Cellular Movement, Haematological System, Immune Cell Trafficking*, *Haematopoiesis*, *Tissue Development*, *Antigen Presentation*, *Cellular Compromise*, *Cellular Function and Maintenance* and *Inflammatory Response*. The T5 networks contained a total of 109 differentially expressed genes involved in pathways and functions including *Inflammatory Response, Digestive System Development and Function, Hepatic System Development and Function, Cell-To-Cell Signalling* and *Interaction, Tissue Development, Haematological System Development and Function, Kidney Failure, Organismal Injury and Abnormalities, Renal and Urological Disease, Tumor Morphology, Amino Acid Metabolism, Small Molecule Biochemistry, Cell Death, Cellular Compromise.*

### Meta-analysis

**Figure 5:**
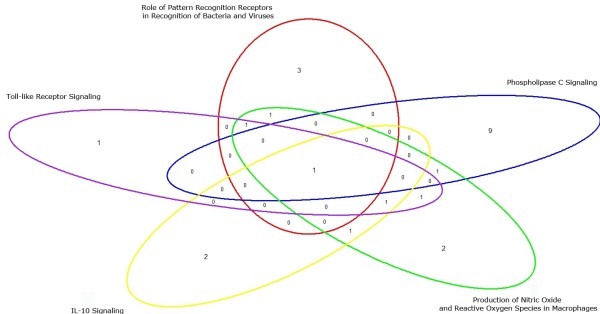
**Venn diagram of the meta-analysis results.** Venn diagram illustrating one gene in common (NFKB1) among the five pathways and distinct genes for the meta-analysis (red: 3 genes of role of pattern recognition receptors in recognition of bacteria and viruses; blue: 9 genes of phospholipase C signalling; green: 2 genes of production of nitric oxide and reactive oxygen species in macrophages; yellow: 2 genes of IL-10 signalling; purple: one gene of toll like receptor signalling).

The meta-analysis of expression data from the milk samples cells across all the time points identified twenty differentially expressed genes (p value <0.0001). These genes could be placed into 5 pathways, with 1 gene (*NFKB1*) in common. Two genes (*TLR2* and *NFKBIA*) were common to 3 pathways: *Toll like receptor signalling*, *role of pattern recognition receptors in recognition of bacteria and viruses*, *production of nitric oxide and reactive oxygen species in macrophages*. The remaining 17 genes were distributed as follows: 9 genes (*HDAC6*, *BTK*, *ARHGEF4*, *RALA*, *RALB*, *FCER1G*, *LYN*, *MYL10* and *ITGA3*) in *phospholipase C signalling pathway*, 2 genes (*IFNGR2* and *IRF8*) in *production of nitric oxide and reactive oxygen species in macrophages*, 2 genes (*IL1RN* and *IL10RB*) in *IL-10 signalling*, 1 gene (*TIRAP*) in *toll like receptor signalling* and 3 genes (*PTX3*, *CLEC7A* and *NOD2*) in *role of pattern recognition receptors in recognition of bacteria and viruses* (Figure 5; Additional file 5).

### Gene expression analysis in White Blood Cells (WBC)

No changes in expression were found between T0 and T1, T2, T3, T4 for WBC either in the HSCS or LSCS lines, or if the data from both lines were combined. However, analysing the combined data, at T5 9 genes were found to be differentially expressed when compared with T0 (p value < 0.05 and log2 fold change > 1.5). Eight of these genes showed an increased level of expression and one showed a decrease in expression (Table 5).

**Table 5:** **List of the 9 differentially expressed genes in white blood cells due to intra-mammary infection with *S. aureus***

**Gene symbol**	**Gene name**	**Gene description**	**Log fold change**
T_5_			
*PTX3*	ENSBTAG00000009012	Pentraxin-related protein PTX3 precursor (Pentaxin-related protein PTX3)	4.9
*DNAJB6*	gnl|UG|Bt#S26165666	Bos taurus DnaJ (Hsp40) homolog, subfamily B, member 6 (DNAJB6), mRNA	2.8
*S100A8*	ENSBTAG00000012640	Protein S100-A8 (S100 calcium-binding protein A8) (Calgranulin-A) (Neutrophil cytosolic 7 kDa protein) (P7) (BEE11)	2.3
*TKDP3*	ENSBTAG00000014345	Trophoblast Kunitz domain protein 3 (Fragment)	2.3
*EMR1*	ENSBTAG00000007901	EGF-like module-containing mucin-like hormone receptor-like 1 precursor (Cell surface glycoprotein EMR1) (EMR1 hormone receptor)	2.3
*ACVR1B*	gb|CO881044.1|CO881044	Homo sapiens activin A receptor, type IB (ACVR1B), transcript variant 1, mRNA	1.7
*TGM1*	ENSBTAG00000003920	transglutaminase 1	1.6
*CD14*	ENSBTAG00000015032	Monocyte differentiation antigen CD14 precursor (Myeloid cell-specific leucine-rich glycoprotein)	1.5
*AMICA1*	ENSBTAG00000023283	AMICA1 protein	−1.6

### Real-time PCR analyses for microarray data validation

The expression of *RPL13A* (Ribosomal Protein L13A) showed no significant variation among time points for both milk and blood cells on the array and was therefore used as reference gene for qPCR for both milk and blood samples.

Real-time qPCR was used to confirm the gene expression differences in expression of some genes between 24 h (T4) and 30 h (T5) post challenge for milk derived cells and 30 h (T5) for blood cells.

The gene with the greatest increase in expression from the array data was the *PTX3* (Pentraxin 3), which showed a logFC of 5.6 in the milk derived cells at T4 and a logFC of 5.0 in blood cells at T5. The strong increase in gene expression of this gene was confirmed by qPCR with a logFC of 8.6 (T4 vs T0) and 8.9 (T5 vs T0) for milk and 7.2 (T5 vs T0) for blood samples.

Changes in expression observed at 30 h after challenge (T5 vs T0), from the microarray data were confirmed by qPCR for following genes: S100 calcium-binding protein A8 (logFC 3.0), hormone receptor-like 1 (logFC 3.0), hormone receptor-like 1 precursor (logFC 3.3) genes for blood and colony stimulating factor 3 receptor (logFC 4.4), Complement C3 precursor (logFC 5.6), IL-18 receptor beta (logFC 5.9), leukocyte elastase inhibitor (logFC 3.9), myeloid differentiation (logFC 3.2), pellino protein (logFC 4.3), T-cell activation Rho (logFC 4.4), Thrombomodulin fragment (logFC 3.9), Toll-like receptor 4 precursor (logFC 5.5), tumor necrosis factor (logFC 6.7) genes for milk samples (Additional files 6 and Additional file 7).

## Discussion

This study used a custom microarray to characterise the changes in gene expression during the early response of the goat mammary gland following intra-mammary infection with *S. aureus*. As insufficient caprine sequences were available to create a dedicated goat array, a bovine Combimatrix 90K custom array was used [23]. Technical replicates in preliminary cross species hybridization experiments showed good correlation between replicates confirming the possibility to successfully use this microarray for gene expression analysis in goats. The potential mis-identification of genes using a cross species array was considered to be a potential problem, however, this could be corrected for using specific probes in a subsequent qPRC confirmation step. Thirteen genes with differential expression pre and post infection identified from the microarray analysis were tested by qPCR. In all cases the direction and magnitude of the change in expression seen on the array was confirmed, thus validating the use of the bovine array with goat.

### Transcriptome differences between high and low SCC animal groups

Somatic cell score is strongly correlated with intra-mammary infection, and mastitis incidence in cattle. Somatic cells score is used in genetic selection with the hope that this will select animal genetically less susceptible to mastitis. Pathogens can only multiply in sufficient quantity to become resident in the gland if the defence mechanisms fail, or are slow to respond to the infection. Therefore, control of invading pathogens within the mammary gland is crucial to prevent the establishment of an infection in the udder, therefore immune cell recruitment is an important part of mammary gland defence against pathogens. Understanding the genetic basis of activation and recruitment of these cells is therefore important to understand the role of these cells in mastitis defence.

Goats from two lines selected for high and low SCS were investigated in this study with the hypothesis that the mammary response and function may differ between the lines. A significant difference in macrophage numbers in the udder was observed between the two divergent selection lines prior to infection (T0). As macrophages are the first cellular type involved in the elimination of invading bacteria, the difference may result in dissimilarities between the HSCS and LSCS in early response to natural infection. However, no differences in gene expression in the milk derived cells between the two lines were observed following the experimental challenge carried out in this study. The changes in the numbers and ratios between of cell types are variables dependent on the individual with as much variation seen between compared within line.

Changes expression were also measured in WBC to examine the type and kinetics of systemic in addition to mammary specific responses. Changes in gene expression were observed in the udder from the earliest time point following infection and at 30 hours post infection a large number of genes showed differences. In the blood white cells (WBC) no change in expression was observed at early time points, while at 30 hours post infection only 9 genes were found to be differentially expressed. As may be expected this shows that the systemic response is slower than in the udder following infection with *S. aureus* and that the changes in expression are more restricted, at least in the early phases. This is not surprising as cells in the udder are becoming activated in themselves to respond directly to the invading pathogen or signalling to activate and recruit other cells to the site of infection. This is seen in the gene pathways that are activated and discussed below. Changes occurring in the blood are more suitable, at least at these early stages of infection. Unfortunately samples were not available to investigate how the response progressed later than 30 hours following infection.

### Gene expression of milk somatic cells

The PMN, lymphocytes, macrophages, and epithelial cells in the milk represent important components of the innate immune defence of the mammary gland [10].

These cells release chemokines that stimulate a rapid influx of PMN to combat the bacteria. This response was clearly seen in the present study such that at 24-30h post infection PMN reached over 80% of cells present, while the percentages of macrophages, lymphocytes and epithelial cells significantly decreased. Differences in cell types composition change the genes products that are detected, irrespective of the actual changes in expression occurring within particular cell types. This makes the interpretation of the data difficult, as the cell population, as well as gene expression within cell types have to be taken into account.

The significant change in macrophage percentages among different time points is related to changes in genes detected that are within canonical macrophages specific pathways such as *MIF-mediated Glucocorticoid Regulation*, *MIF Regulation of Innate Immunity*, and *Production of Nitric Oxide and Reactive Oxygen Species in Macrophages.* The expression of these pathways increased in the context that the macrophage cells percentage decreased, then there is an enormous increase in expression of these genes. Therefore, the microarray data from milk SCC in this study partly reflect changes in gene expression as a result of alteration in cell proportions within the mammary gland, and partly changes in expression within cell types.

At 24 h following inoculation with *S. aureus* transcription levels of 300 genes were significantly changed in milk somatic cells. These genes fell into functional pathways which associated with cell death, cellular movement, cellular growth and proliferation, cell-to-cell signalling and interaction, and lipid metabolism.

The levels of the pro-inflammatory cytokines tumour necrosis factor alpha (*TNFα*), interleukin 1 alpha (*IL-1α*), interleukin 8 (*IL-8*) were increased in the SCC following challenge. These cytokines are associated with inflammatory processes and are involved in recruitment and activation of neutrophils into the infected tissue [18]. This response following the recognition of invading pathogens, is characteristic of macrophages present among the milk somatic cells, and is instrumental in attracting PMN involved in immune defence from the blood to the mammary gland. The response to this signalling is observed in the increased % of PMN found in the infected gland at 30 hours. Expression of the gene coding for CD14 antigen (*CD14*), a pivotal cell surface protein on macrophages, that interacts with toll-like receptors mediating detection of bacterial cell wall components, was also increased.

The acute phase response is a rapid and a non-specific inflammatory response that provides the initial line of protection against microorganisms and is regulated through the expression of several cytokines [24]. This is consistent with the early stage response observed in the SCC which involves changes in expression of pathways, including *IL-10 Signalling* (T4) and *LXR/RXR activation* (T5). A close relationship between polyamine regulation, in particular the sub-group spermine, and *IL-10 Signalling* has been previously reported in macrophages [25]. An increase of *IL-6* and *IL-10* expression in somatic cells during mastitis infection has also been previously reported in cattle [18,26].

The persistence inflammation, induced by cytokines, is harmful for cells [27] and the affected organ. Therefore the observed activation of the *IL-10 Signalling,* which modulates immune response during the early acute response stage of mastitis infection is important to limit the inflammatory response and tissue damage within the udder. Interleukin-10 is an anti-inflammatory cytokine that blocks NF-kB activity, which leads to suppression of pro-inflammatory mediators such as TNF, IL6, and IL1. Expression was significantly increased for 7 out of 78 putative genes of the *IL-10 Signalling* pathway present on the microarray following infection. Interleukin 1 receptor antagonist (*IL1RN*) and interleukin 10 receptor beta (*IL10RB*) are also part of the *IL-10 Signalling* pathway and limit inflammatory response, as well as regulating B-cells, T-cells, NK-cells and dendritic cell division and differentiation, which is associated with the immune response.

Lipid metabolism is generally inhibited during the intra-mammary infections [10]. In the present study the *LXR/RXR* pathway which is involved in the regulation of lipid metabolism, inflammation, and cholesterol to bile acid catabolism [28] showed reduced expression in SCC following infection. The *PPAR* family consists of *PPARα*, *PPARδ*, and *PPARγ*, each of which act as ligand activated transcriptional regulators. Expression of these genes was found to be reduced following infection in the present study. The *PPAR* ligands include n-3 and n-6 unsaturated fatty acids and their eicosanoid products, thus PPARs are involved in the regulation intracellular lipid levels. *PPAR* is highly expressed in adipose tissue and macrophages and primarily regulates adipogenesis, but also interferes with the transcription of proinflammatory factors such as STAT and NF-κB in macrophages [29]. The down regulation of LXR and RXR and the PPAR genes suggest that during the early stage response to mastitis there might be a “general” deregulation of the lipid metabolism. This has also been observed in cattle infected with *S. uberis* by Moyes *et al.*[19], where the intra-mammary infection led to the activation of pro-inflammatory pathways, and a marked inhibition of lipid synthesis. The expression of LXR and PPAR and signalling most likely by PPAR*γ,* may provide mechanistic explanation for the inverse relationship between immune response ad fat synthesis. In humans, there is evidence that lipoproteins are part of the innate immune system, and changes in lipid and lipoprotein metabolism that occur during the host response to infection have an anti-infective and anti-inflammatory effect that contributes to the host defence [30].

Moreover, in the stromal vascular fraction, adipose tissue also contains macrophages that secrete pro-inflammatory cytokines, such as TNF-α, IL-1β and IL-6 [31]. Studies focused on the effect of intra-mammary infection in adipose tissue of ruminants are lacking. In a recent work adipose tissue in dairy cows appears to respond *in vitro* to an immune challenge by increased gene expression of cytokines [32].

Meta-analysis approaches are used to combine or integrate information from independent studies to overcome low statistical power in studies and identify common results to increase confidence in the generality of information [22]. In the present study meta-analysis was used to combine information across samples and time-points, which identified 17 differentially expressed genes (Figure 5) which are involved in five pathways. Pentraxin 3 (*PTX3*) was significantly associated with response to infection and increased the expression in both SCC and blood PMN. PTX3 is a C-type lectin domain family 7 member A (*CLEC7A*) and nucleotide-binding oligomerization domain containing 2 (*NOD2*) belonged to the *Role of Pattern Recognition Receptors in Recognition of Bacteria and Viruses* pathway.

The innate immune system uses a several receptors (*PRRs*) that recognize conserved microbial structures or pathogen-associated molecular patterns (*PAMPS*), such as those that occur in the bacterial cell-wall components peptidoglycan and lipopolysaccharide. *PPRs* are classified as: (i) membrane bound *PRRs* which include toll-like receptors (TLRs), (ii) extracellular *PRRs* that include the complement proteins, pentraxins and collectins involved in the process of opsonization, phagocytosis and apoptosis and (iii) cytoplasmic *PRRs* which include the *NOD* proteins that regulating the cytokine induction initiated by bacterial ligands.

*PTX3* is involved in the initial response of mammary tissue to bacterial infection [18,33,34], and is thought to activate the immune system by binding both micro-organisms and C1q, the first component of the classical pathway of complement activation. *NOD2*, is expressed in monocytes/macrophages, and detects peptidoglican from gram-negative (for example *E. coli*) and gram-positive bacteria (*S. aureus*) [35,36]. Although *S. aureus* is usually an extracellular bacterium, it has been shown to be internalised by different mammalian cells such as pulmonary epithelial cells, enterocytes and neutrophils in a manner dependent on the expression of the virulence regulators *Agr* and *SarA*[37]. The peptidoglycan fragments from cytoplasm-dwelling bacteria could, therefore, be available for recognition by *NOD2* and initiate Nod-dependent cellular responses, making this gene a potential marker for this pathogen infection.

### Gene expression white blood cells

The first changes in gene expression seen in WBC were observed at 30h post-infection and coincided with the lowest TBL and NEU counts. At this time point 9 genes were found to be differentially expressed. As for milk somatic cells, in WBC the gene with the highest fold change was *PTX3*. It was the first long pentraxin identified, an interleukin-1 inducible gene expressed in endothelial cells [38] and is a tumor necrosis factor α (*TNF-α*) inducible gene in fibroblasts [39]. *PTX3* is produced by macrophages and other cell types upon stimulation with primary inflammatory mediators such as LPS, IL-1 or TNF-α [40]. *PTX3* plasma levels are very low in normal circumstances (≤2ng/ml) but expression has been shown to increase following pathogen infection [41]. *PTX3* is a functional ancestor of antibodies, recognizing microorganisms, activating complement, and facilitating pathogen recognition by phagocytes. Hence it plays a role in resistance against selected pathogens [42]. Mature *PTX3* is stored in specific granules by neutrophils and is secreted following recognition of microbial moieties and inflammatory signals [43]. This protein has been previously identified as one of the most up-regulated genes after IMI with *S. aureus*[18]. *PTX3* together with *S100A12* act as anti-microbial agents that could assist defence of the mammary gland against chronic and subclinical infections. In the present study *PTX3* was the most significantly up-regulated gene in both somatic cells (T4 and T5) and in WBC (T5) in response to *S. aureus* infection in goats.

## Conclusions

In the present study early response to an experimental challenge with *S. aureus* was studied between two divergent lines of goat selected for high and low milk somatic cell scores. Although the two lines had a pre-infection difference in milk SCC, the gene expression analysis of SCs from the two lines (HSCS vs LSCS) following infection did not show significant differences. The expression analysis showed that expression changes were largely consistent with changes in the cell types in milk, and the activation of immune cell types. An inverse relationship between immune response and lipid metabolism was observed and has previously been described in other species, including cow and sheep.

*PTX3* had the higher log fold change in expression in both in milk and in blood and is a candidate for further studies on the genetic basis of variations to in immune response associated with mastitis.

## Methods

### Animals

Two groups of five primiparous goats from divergent selection for extreme breeding values for the somatic cell score (one group from the high SCS line and the other from the low SCS line) were selected to have similar milk production (3.2 ± 0.5 kg/d) and provided by INRA. Basically, an experimental genetic evaluation was performed using data from 140,000 primiparous alpine goats in 3625 flock by year combination. The trait considered was the lactation mean of monthly somatic cell scores (LSCS) as detailed in *Rupp et al*. [12]. Breeding values for LSCS were expressed in genetic standard deviation with inverted sign (positive index are favorable). Two groups of 5 and 6 bucks with extreme high and low breeding values for LSCS (−1.38 ± 0.68 vs 1.17±0.48, respectively) were selected to sire daughters at the INRA experimental facility of Bourges (UE0332, OSMOY, France). Out of 52 born kids, two groups of 5 daughters (sired by 3 and 4 high and low SCC bucks, respectively) were transferred two months before first kidding to “Centro Zootecnico Didattico Sperimentale dell’Università degli Studi di Milano-Italy” (http://www.veterinaria.unimi.it/Facolta/2586_ITA_HTML.html) for experimental infection.

The study was carried out when the goats were of first parity and at the peak of lactation (48 ± 2 days in milking). Goats were monitored for intra-mammary infections (particularly for *S. aureus*) from parturition to the day of challenge with bacteriological analysis and somatic cell scores measured on milk samples each week as described by *Pisoni et al*. [21] and *Moroni et al.*[44]. Fore milk samples were collected on the three days immediately prior to the experimental infection and all animals were shown to be free of any mastitis pathogens and to have SCC below 250,000 cells/mL.

All experimental procedures were performed according to the Italian legislation, following approval by the ethics committee of University of Milan.

### *Staphylococcus aureus* strain

*S. aureus* strain DV137, which was originally isolated from chronic case of caprine mastitis [45], was used for the experimental infections. *S. aureus* DV137 is positive for clumping factor, free coagulase, enterotoxins C and L, toxic shock syndrome toxin TSST-1 and leukocidin LukDE.

The *S. aureus* strain DV137 was grown from an individual colony by transfer to 10 mL of brain heart infusion broth (Becton-Dickinson Diagnostic Systems, Inc., Milan, Italy) and incubated for 6 h at 37°C. Thereafter, 1 mL of the culture was transferred to 99 mL of tryptic soy broth (TSB, Difco, Milan, Italy) and incubated overnight at 37°C. The concentration of the bacterium in this stock culture was maintained at 4°C overnight. Before infection the stock was diluted with sterile pyrogen-free Phosphate Buffered Saline (PBS, Invitrogen, Milan, Italy) to give a final concentration of 10^3^ Colony Forming Unit/mL (CFU/mL) for the experimental infection.

### Intra-mammary challenge

Prior to intra-mammary challenge the goats were milked by hand and their udders emptied; the teat ends were carefully disinfected with chlorhexidine. The left udder half of each goat was infused with 1 mL (10^3^ CFU/mL) inoculum of *S. aureus*. The right udder half was infused with 1 mL of sterile pyrogen-free PBS. Inoculation was administered intra-cisternally through the teat canal using a sterile blunt needle. Milk samples were collected from all goats from both udder halves just prior to the challenge (T0) and at 6 (T1), 12 (T2), 18 (T3), 24 (T4) and 30 (T5) hours after the experimental infection. At each time point each goat was recorded for: general demeanour, stance, position, food/water intake and vocalization, temperature and the udder examined for temperature, swelling, colour, pain, lumps, injuries to the teats/udder, milk letdown, milk colour and milk clots. A paired t-test (with threshold for statistical significance set to 0.05) was applied to body temperature of LSCS and HSCS to test if the differences observed at each time point were significant.

### Bacteriology and milk somatic cells analysis

Twenty ml milk samples were taken under strictly hygienic conditions from each udder half into two sterile 10 ml tubes. One of the tubes was used for bacteriology, while the other tube of milk was used for SCC analysis. Subsequently, 200 mL of milk were collected from the left infected udders for isolation of cells and further analysis. A paired t-test (with threshold set to 0.05) was applied to milk SCC of right udders in LSCS and HSCS goats to test if the changes at each time point were significant.

Milk SCC were determined in 2-mL of milk, which was heated for 15 min up to 60°C then maintained at 40°C until analysed, in duplicate, on automated fluorescent microscopic somatic cell counter (Bentley Somacount 150, Bentley Instrument, Milan, Italy). For differential cell counts (DCCs), 10 ml of milk were centrifuged for 15 min at 400 *x g*, the cream layer and supernatant were discarded and the cells were washed twice in PBS. DCCs of isolated cell suspensions were estimated by means of esterase stain [46]. Evaluation of the slides was carried out using light microscopy and oil immersion (100-fold magnification). One-hundred cells of each slide were counted meander-shaped and defined as lymphocytes, macrophages, PMN and epithelial cells. Cell identification was achieved using standard methods [47,48].

Aseptically collected milk samples were diluted in sterile PBS and plated onto blood agar plates. The number of CFU was determined after 16 h of incubation at 37°C. Colonies displaying haemolysis were initially counted as *S. aureus*, and subsequently confirmed microscopically and biochemically by the presence of Gram-positive cocci that were both catalase- and coagulase-positive. *S. aureus* isolates were tested by RAPD-PCR [49] to confirm that they were the strain used in the experimental challenge.

### Blood sample analysis

Ten ml of blood samples were collected into EDTA from the jugular vein just prior to the challenge and 6, 12, 18, 24, and 30 hours after inoculation.

Blood samples were analyzed using the ADVIA 120 (Siemens Healthcare) haematology system and the multispecies software provided by the manufacturer. The following parameters were calculated: erythrocyte count (RBC), haemoglobin concentration, haematocrit, mean corpuscular volume (MCV), mean corpuscular haemoglobin (MCH), mean corpuscular haemoglobin concentration (MCHC), leukocyte count (WBC), platelet count (Plt), mean platelet volume (MPV). Leukocyte different counts (percentage and number of neutrophils, lymphocytes, monocytes, eosinophils, basophils and large unstained cells or LUC), were also performed. A paired t-test (with threshold set to 0.05) was applied to blood sample parameters of LSCS and HSCS animals to test if differences at each time point were significant.

### Isolation of white blood cells (WBC)

Ten ml of fresh blood were transferred into a 50 ml tube and 20 ml of 0.2% NaCl were added, mixed with 5 ml of NaCl 3.7% and then centrifuged at 1000 x g for 10 min at 4°C. The pellet was resuspended in 15 ml of PBS and the solution was centrifuged at 1000 x g for 10 min at 4°C. The supernatant was discarded and the pellet was resuspended in 1 ml of Trizol (Invitrogen, Milan, Italy).

### Preparation of milk somatic cells

Fore milk was collected aseptically from both halves of the udder of each goat prior to the challenge and 6, 12, 18, 24, and 30 hours post-infection. Fifty mL of milk were transferred to falcon tubes and centrifuged at 750 x *g* at 4°C for 10 min. The fat layer and the supernatant were discarded and the cell pellet was re-suspended and washed in PBS pH 7.2. After a centrifugation at 450 x *g* for 10 min, the supernatant was discarded and the pellet was resuspended in 3 mL of Trizol (Invitrogen, Milan, Italy).

### RNA extraction

From WBC and somatic cells total RNA was extracted following the instructions of the supplier (Invitrogen, Milan, Italy), further purified using an RNeasy MinElute spin column (Qiagen, Milan, Italy) and eluted in RNase-free water. RNA was quantified using a NanoDrop spectrophotometer (NanoDrop Technologies, Wilmington, DE, USA) and quality-checked using a Bioanalyser 2100 (Agilent, Santa Clara, CA). RNA samples with RNA Integrity Number (RIN) values between 7.0 and 10.0 were used for the microarray analysis.

### Microrray design

The microarray design has been described previously [23], briefly all available bovine transcript sequence information was downloaded from Ensembl release 50, and Unigene and dbEST databases (Sept. 2008). A bioinformatic pipeline was created to align the sequences and select a unique set of minimally redundant bovine transcripts. This dataset was used to design 43,768 unique probes with a length of 35 nucleotides, each representing a single bovine transcript. The microarray probes were designed at the 3′ end of the bovine sequences. These probes were synthesised in duplicate, along with negative and quality controls, on a 90K feature custom array from CombiMatrix (Seattle, WA).

### Array hybridization and statistical analysis

One μg *RNA* was amplified and labelled with Cy5-ULS using the *RNA* Amplification and Labelling Kit from CombiMatrix (ampULSe Cat. no. GEA-022; Kreatech Biotechnology, Amsterdam, The Netherlands). All procedures were carried out according to the manufacturer’s protocols. The purified labelled *aRNA* was quantified using a NanoDrop spectrophotometer (NanoDrop Technologies, Wilmington, DE, USA). Four μg of labelled *RNA* were fragmented to a uniform size and hybridized to the custom array following the Combimatrix CustomArray 90K Microarray Hybridization and Imaging Protocol Arrays were stripped and re-hybridised using the CustomArray Stripping Kit for 90K (CombiMatrix Cat. No. 610049) following the protocols of the manufacturer. Each array was used up to 4 times with no deterioration in signal or increase in background.

To verify the powerful of this bovine custom array for the caprine gene expression study, a series of technical replicates were carried out showing a signal intensities correlation higher than 97% (data not shown).

The hybridised arrays were scanned with a GenePix 4000B microarray scanner (Axon, Toronto, CA) and the images (TIF format) were exported to the CombiMatrix Microarray Imager Software, to perform quality checks of the hybridizations and the spots on the slide. Data were extracted and loaded into the R software using the Limma analysis package from Bioconductor. A design matrix was created using Limma functions to describe the experimental samples and replicates. The raw intensities were processed using quantile normalization and data were then transformed in log2 to be used for the statistical analysis.

Limma performs a linear regression analysis on the hybridizations, using a group-means parameterization approach to compare the different conditions and performs a false discovery rate adjustment with Benjamini-Hochberg correction for multiple testing [50]. False discovery rates of 1% and 5% were accepted for SC and WBC respectively and DE genes were selected using an adjusted P-value cut off equal to 0.01 and 0.05.

The microarray data files have been deposited in NCBI’s Gene expression Omnibus (GEO; http://www.ncbi.nlm.nih.gov/geo/) at the identifier number GPL14856 and the experiment at the identifier number GSE33894.

### Assignment of affected genes to pathways, networks and biological functions

Each gene symbol of the affected genes identified with R was mapped to its corresponding gene object in the Ingenuity Pathways Knowledge Base. Feeding the lists of affected genes as input to the IPA library identified associated canonical pathways, biological functions and networks which were used to investigate the biological context.

The IPA library items were ranked based on significance of association with the input list of genes. For the canonical pathways this significance was determined based on two parameters: (i) ratio of the number of genes from the input data set that map to the canonical pathway divided by the total number of genes of that pathway and (ii) p-values calculated using Fischer’s exact test determining the probability that the association is explained by chance alone.

For the biological functions and networks the significance was linked to the p-value only, calculated by right-tailed Fisher’s exact test. The p-values for the network analysis take into account the number of affected genes in the network and the size of the network.

### Meta-analysis procedures

Data from all time points were loaded into R software, the signal intensities were processed and normalized using standard Limma procedures, then all the expression data were put together.

MetaMA package was used to perform a moderated t-test with a Benjamini Hochberg (BH) correction at a 0.01 % threshold (to take into account the multiple testing problem) to each time point compared with T0. Once obtained, the p-values from study-specific analysis we combined them into one p-value in sense of sum of logs.

The Venn diagram was built using a modified version of R script “Venn”.

http://bioinfo-mite.crb.wsu.edu/Rcode/Venn.R.

### Real time PCR validation

For the optimization of all real-time assays, ten milk RNA samples at T0 were pooled and normalized to a final concentration of 200 ng/μl and the same procedure was used for samples from T4 and T5. RNA from 10 WBC samples at T0 were pooled and normalized to a final concentration of 100 ng/μl and the same procedure was applied to T5 samples. Pooled samples were serial diluted and used for the set-up of the standard curve (5 points in triplicates); in each assay a negative control was also included.

Samples were analysed one by one: 1 μg of milk and 0.5 μg of blood samples were individually reverse-transcribed using the Superscript II RT-PCR System (Invitrogen Life technologies) following the manufacturers instructions. Each sample was tested in triplicates.

Eleven genes from the milk samples and four from blood samples showing high FC and p-values different between post (T4, T5) and pre-infection (T0), were selected for quantitative real-time PCR validation of microarray results.

Caprine specific rtPCR primers were designed using goat sequence where this was available. When goat sequence was not available transcript-specific primers were designed for the conserved regions of the bovine sequence using Primer Express software (version 3.0) running standard settings.

*ACTB* (actin β), *GAPD* (glyceraldehyde 3-phosphate dehydrogenase), *HMBS* (Hydroxymethylbilane synthase), *RPL13A* (Ribosomal protein L13a), and *YWHAZ* (Tyrosine 3-monooxygenase/tryptophan 5-monooxygenase activation protein, zeta polypeptide) were selected from literature [51] and tested in order to choose a reference gene common to milk and blood analyses according to the variation in the array data. Control cDNA dilution series were created for each gene to establish a standard curve; all real-time reactions were performed in triplicate.

The real-time reaction mixture, in a final volume of 10 μl, included 2 μl dilution 1:10 of the cDNA as template, 1X Power SYBR Green Master Mix (Applied Biosystems, Foster City, CA, USA) and 0.5 μM of each primer forward and reverse (Additional files 6 and Additional file 7) except for blood-gene egf-like module containing, mucin-like, hormone receptor-like 1, where final concentration was 0.05 μM for primer forward and 0.9 μM for primer reverse. The real-time PCR reaction set up was made in 384 optical well plates with a Freedom Evo Robot (Tecan) and carried out on an ABI 7900HT Fast Real-Time PCR System (Applied Biosystem) with a standard programme (50°C*2’/95°C*10’/40 cycles 95°C*15” and 60°C*1’). Data were analyzed with the GeneAmp 7900HT sequence detection system software (PerkinElmer Corp., Foster City, USA).The log input amount of the standard curve was plotted versus the output Ct values and the log input amount of each sample was calculated according to the formula (Ct - b)/m, where b is the Y-intercept, and m is the slope. The log input amount was converted to input amount according to the formula 10^(log input amount), and triplicate input amounts were averaged for each sample.

Data were imported and processed into R using the ddCt analysis package from Bioconductor, time point T0 and *RPL13A* were set as reference sample and reference gene respectively and the 2-ΔΔCt algorithm was applied to find the relative level expression [52].

## Abbreviations

SCS: Somatic Cell Score; SCC: Somatic Cell Count; HSCS: High Somatic Cell Score; LSCS: Low Somatic Cell Score; CFU: Colony Forming Unit; RAPD: Random Amplified Polymorphic DNA; MIF: Macrophage Migration Inhibitory Factor; LXR/RXR: Liver X Receptor/Retinoid X Receptors; PPAR: Peroxisome Proliferator-Activated Receptor.

## Competing interests

In the past five years the authors did not receive any reimbursements, fees, funding, or salary from an organization that may in any way gain or lose financially from the publication of this manuscript. The authors did not hold any stocks or shares in an organization that may in any way gain or lose financially from the publication of this manuscript. The authors did not hold or apply for any patents relating to the content of the manuscript and they did not have financial competing interests.

## Authors’ contributions

PC and RC prepared RNA for hybridization, performed the microarray experiments, the RT-qPCR analysis and drafted the manuscript. RC, PM1 and GP extracted RNA. GP, PM1 and PM2 performed the challenge, collected samples and carried out the bacteriology and phenotypical analyses. MDC and AS performed all the statistical analyses for microarray and meta-analysis. FS designed the microarray and analysed the raw data. RR selected and provided the animals and HC took care of them at the INRA experimental facility. JLW was involved in array design and participated in writing the paper. BC collaborated in microarray experiments and drafted the paper. AS and RR designed the experiment. AS supervised the experimental study. All authors read and approved the final manuscript.

## Supplementary Material

Additional file 1 **Significativity of the differential cell counts analysis calculated for each group of goat (HSCS and LSCS) at different time points.** (XLS 25 kb) Click here for file

Additional file 2 **List of the differentially expressed genes between T4 and T0 in milk samples.** Data were extracted and loaded into R software using the Limma analysis package from Bioconductor and the signal intensities were processed and normalized using standard procedures. Genes were considered as differentially expressed if p value < 0.01 and log2 fold change > 1.5. Click here for file

Additional file 3 **List of the differentially expressed genes between T5 and T0 in milk samples.** Data were extracted and loaded into R software using the Limma analysis package from Bioconductor and the signal intensities were processed and normalized using standard procedures. Genes were considered as differentially expressed if p value < 0.01 and log2 fold change > 1.5. Click here for file

Additional file 4 **Most significant affected IPA canonical pathways.** The most significant pathways are: *MIF-mediated Glucocorticoid Regulation*, *MIF Regulation of Innate Immunity*, *NF-kB Signalling*, *IL-10 Signalling* and *Hypoxia Signalling in Cardiovascular System* for T4 and *Production of Nitric Oxide and Reactive Oxygen Species in Macrophages*, LXR/RXR *Activation, Toll-like Receptor Signalling*, *Acute Phase Response Signalling* and *MIF-mediated Glucocorticoid Regulation* for T5. Click here for file

Additional file 5 **Role of pattern recognition receptors in recognition of bacteria and viruses pathway.** Canonical pathway performed with IPA Knowledge Base. Click here for file

Additional file 6 **Oligonucleotide sequences and results for milk samples q-PCR.** The sequences of the couples of primers are listed in the table with q-PCR results. Click here for file

Additional file 7 **Oligonucleotide sequences and results for blood samples q-PCR.** The sequences of the couples of primers are listed in the table with q-PCR results. Click here for file
