# Supplementary material for: Response of the goat mammary gland to infection with Staphylococcus aureus revealed by gene expression profiling in milk somatic and white blood cells
Source: BMC Genomics. 2012 Oct 9;13:540. doi: 10.1186/1471-2164-13-540 (PMC3532242; doi:10.1186/1471-2164-13-540)
Supplement: Additional file 4 — Most significant affected IPA canonical pathways. The most significant pathways are: MIF-mediated Glucocorticoid Regulation, MIF Regulation of Innate Immunity, NF-kB Signalling, IL-10 Signalling and Hypoxia Signalling in Cardiovascular System for T4 and Production of Nitric Oxide and Reactive Oxygen Species in Macrophages, LXR/RXR Activation, Toll-like Receptor Signalling, Acute Phase Response Signalling and MIF-mediated Glucocorticoid Regulation for T5. [file 1471-2164-13-540-S4.pdf]

MIF-mediated Glucocorticoid Regulation

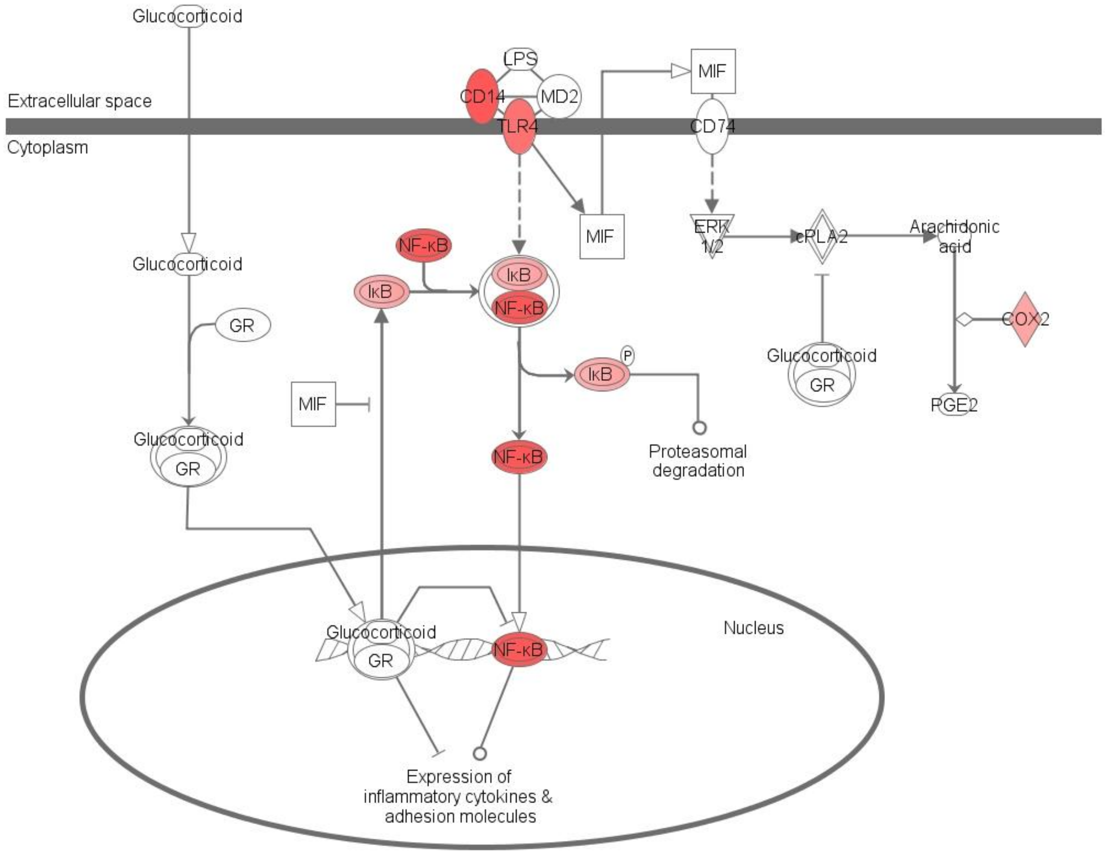

© 2000-2011 Ingenuity Systems, Inc. All rights reserved.

MIF Regulation of Innate Immunity

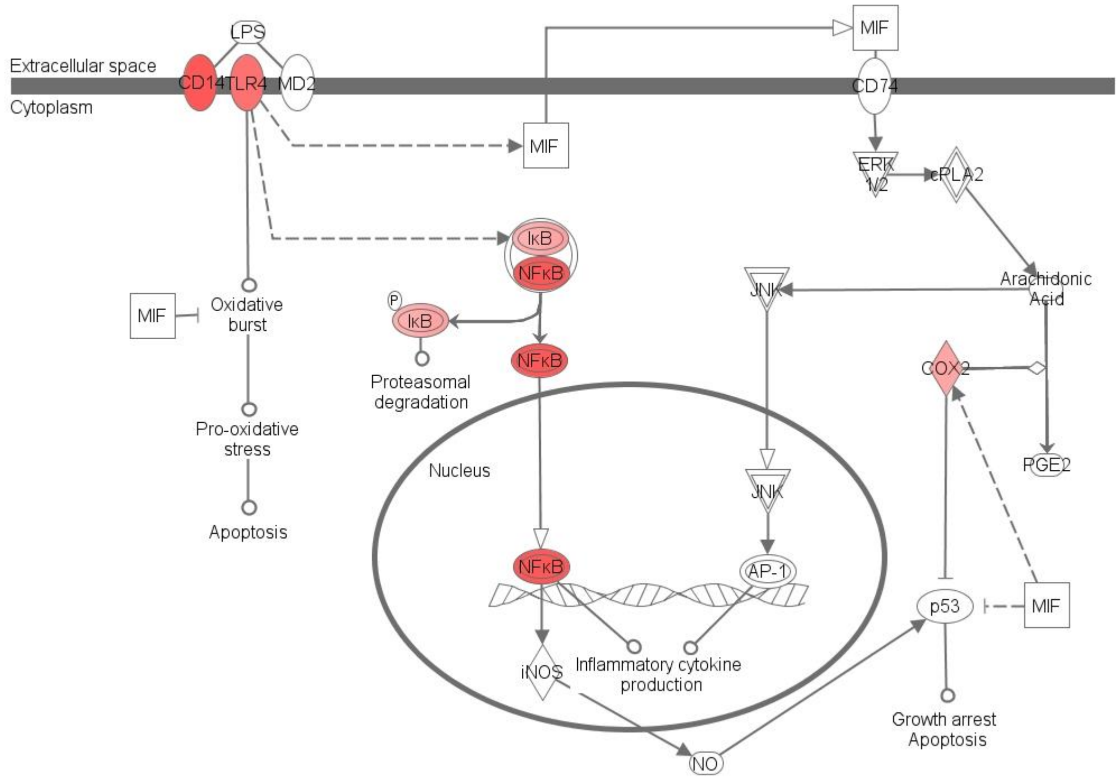

© 2000-2011 Ingenuity Systems, Inc. All rights reserved.

NF-κB Signaling

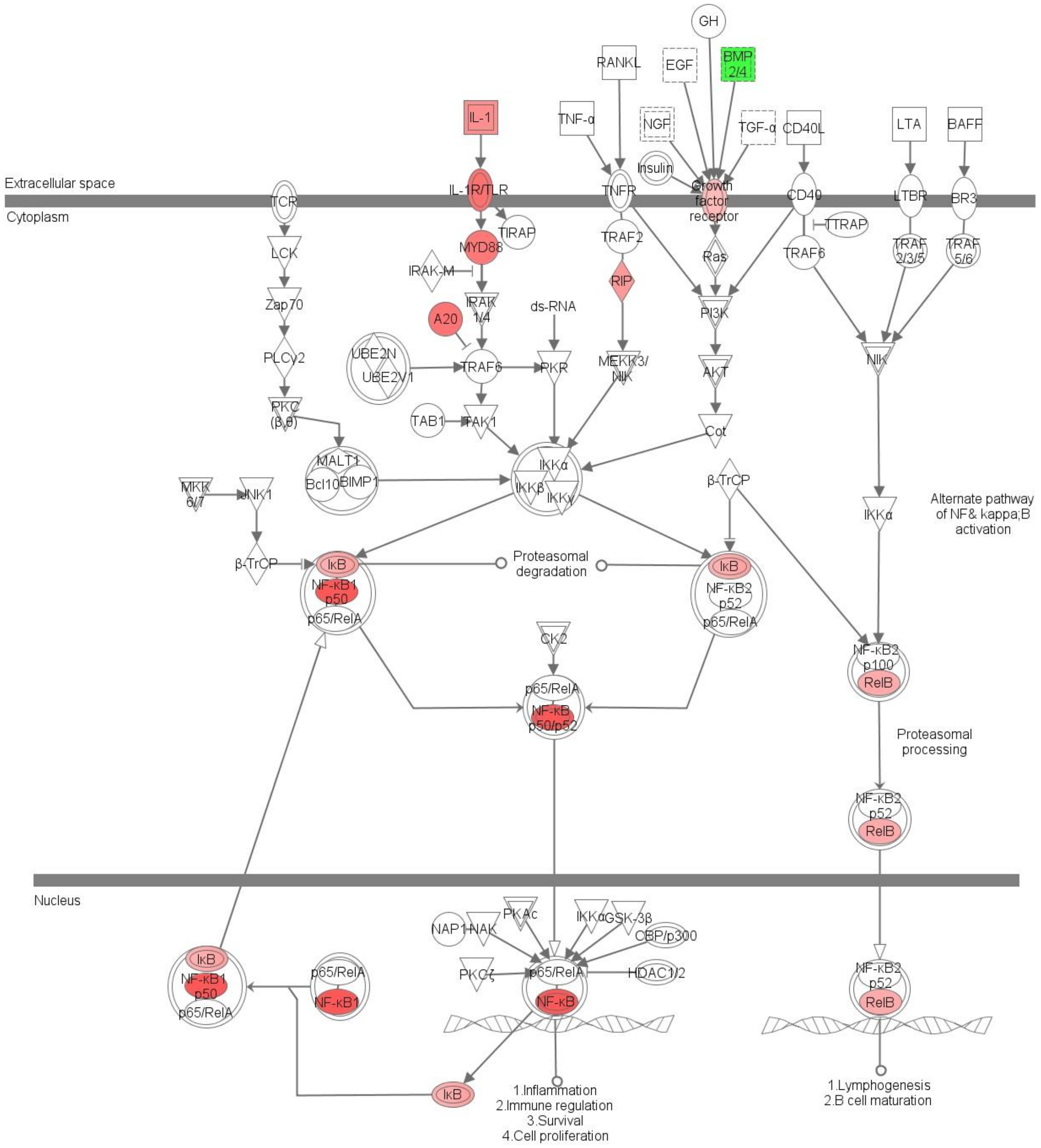

© 2000-2011 Ingenuity Systems, Inc. All rights reserved.

IL-10 Signaling

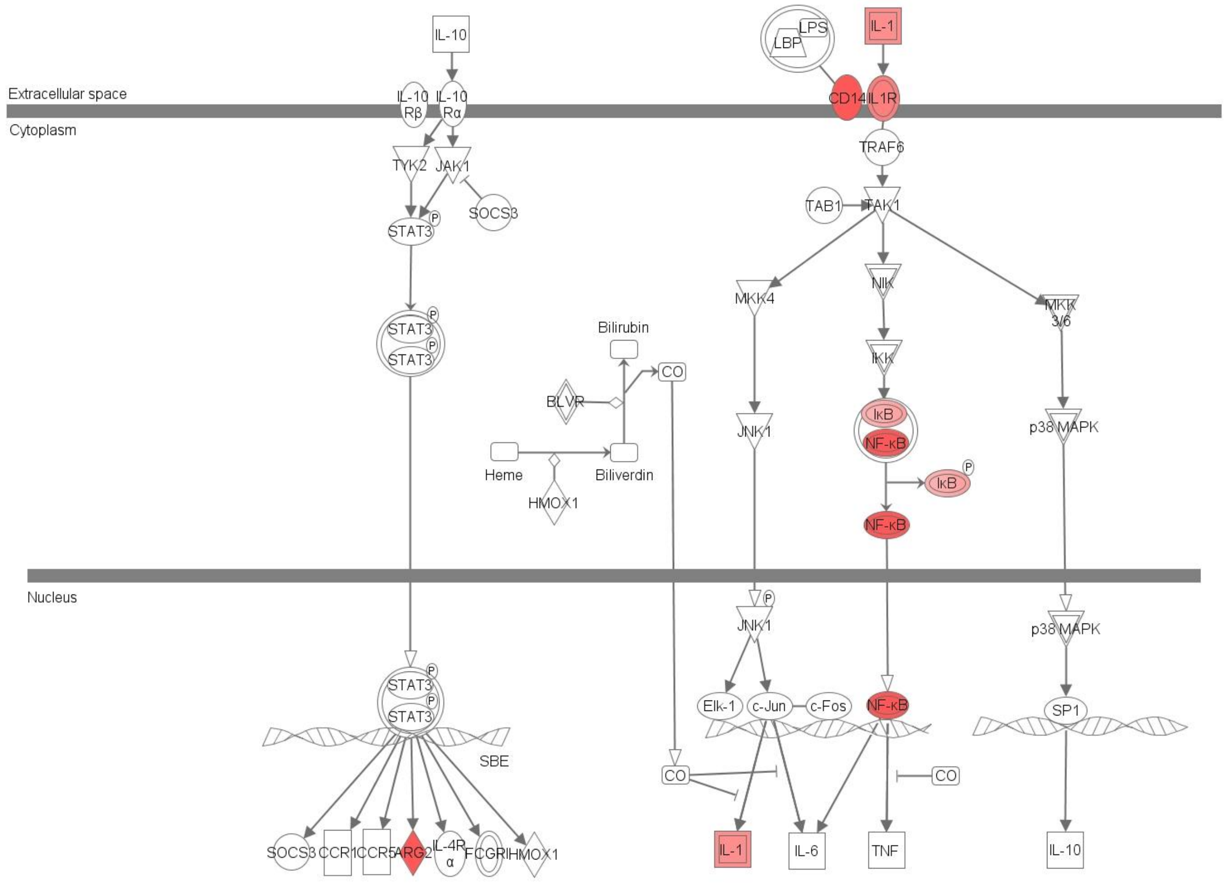

© 2000-2011 Ingenuity Systems, Inc. All rights reserved.

Hypoxia Signaling in the Cardiovascular System

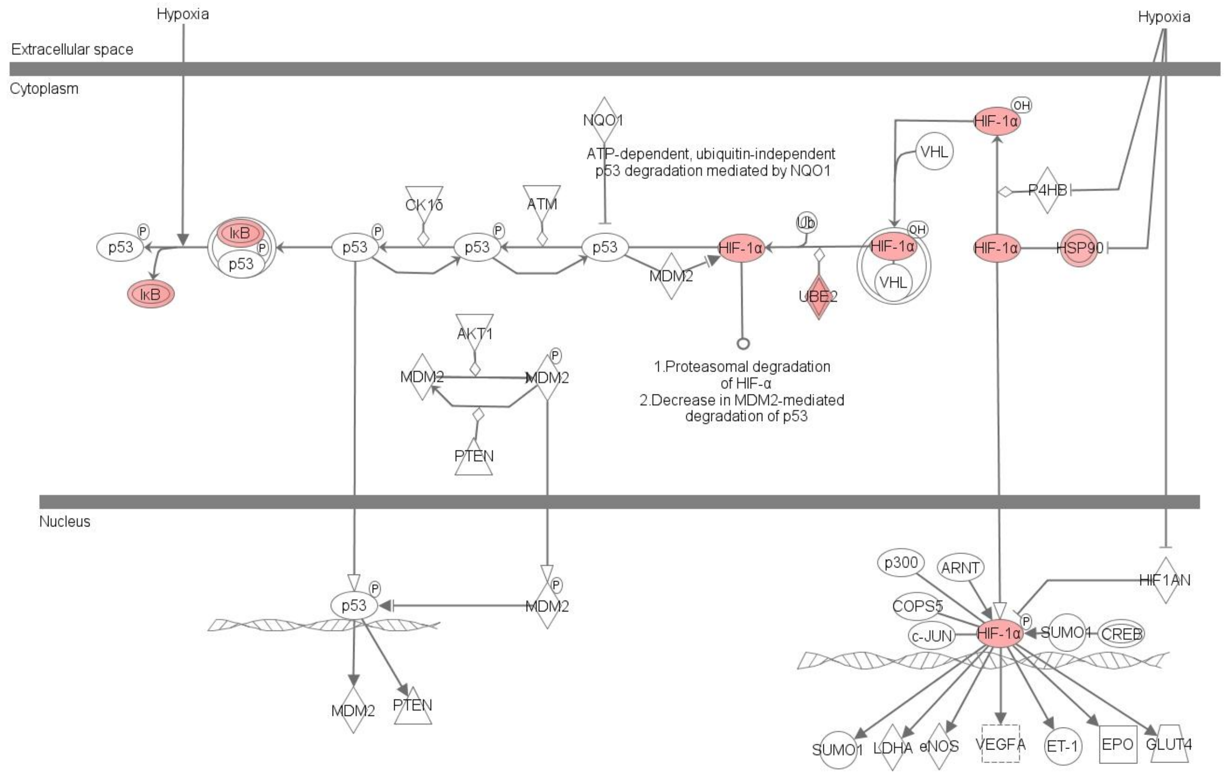

© 2000-2011 Ingenuity Systems, Inc. All rights reserved.

Production of Nitric Oxide and Reactive Oxygen Species in Macrophages

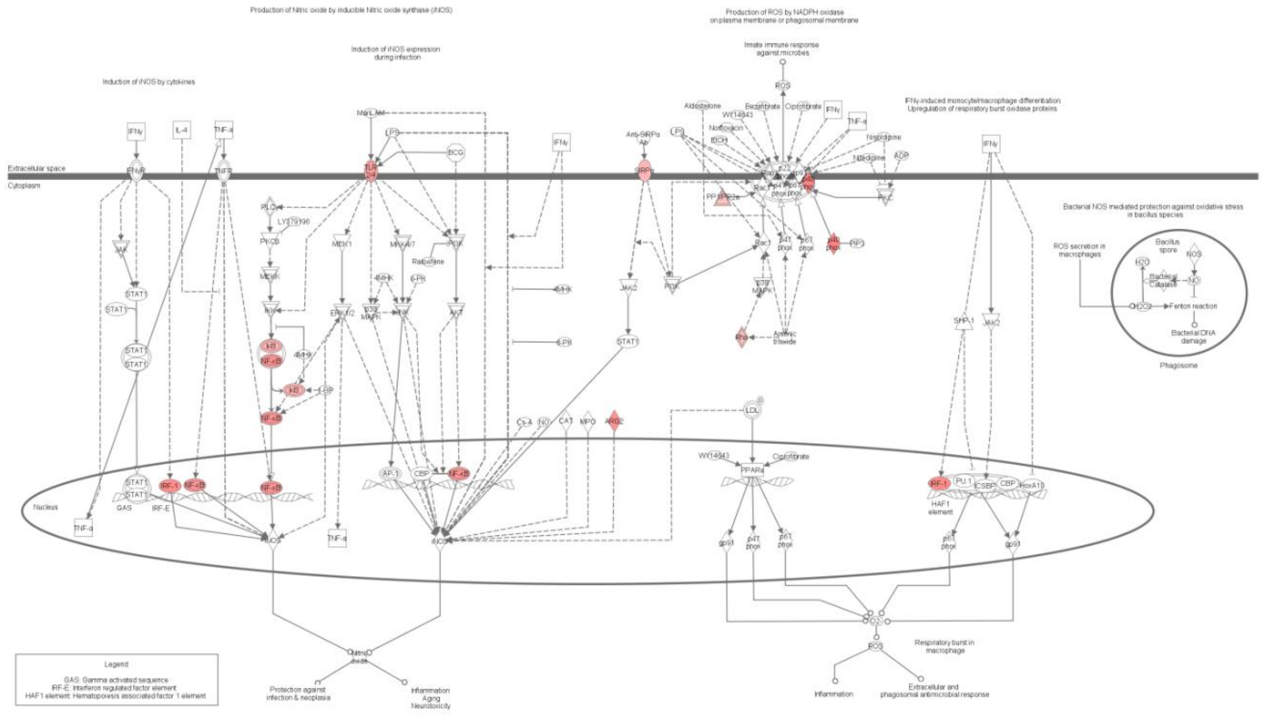

© 2000-2011 Ingenuity Systems, Inc. All rights reserved.

LXR/RXR Activation

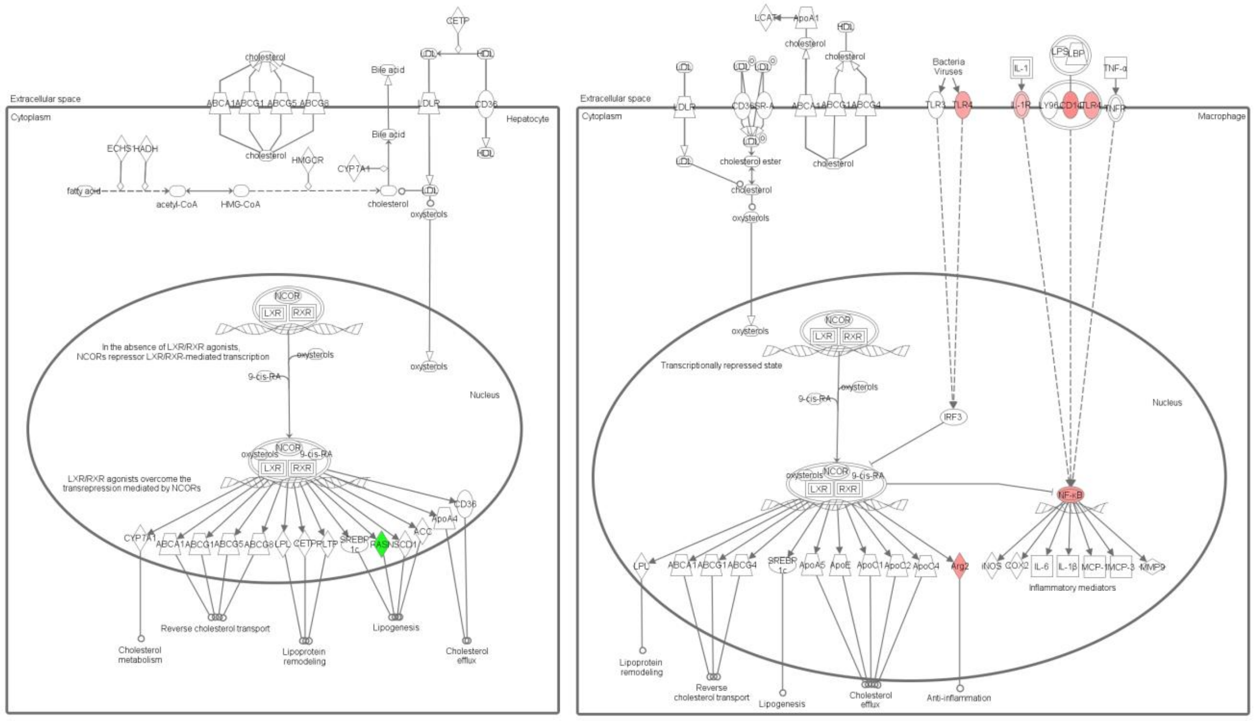

© 2000-2011 Ingenuity Systems, Inc. All rights reserved.

Toll-like Receptor Signaling

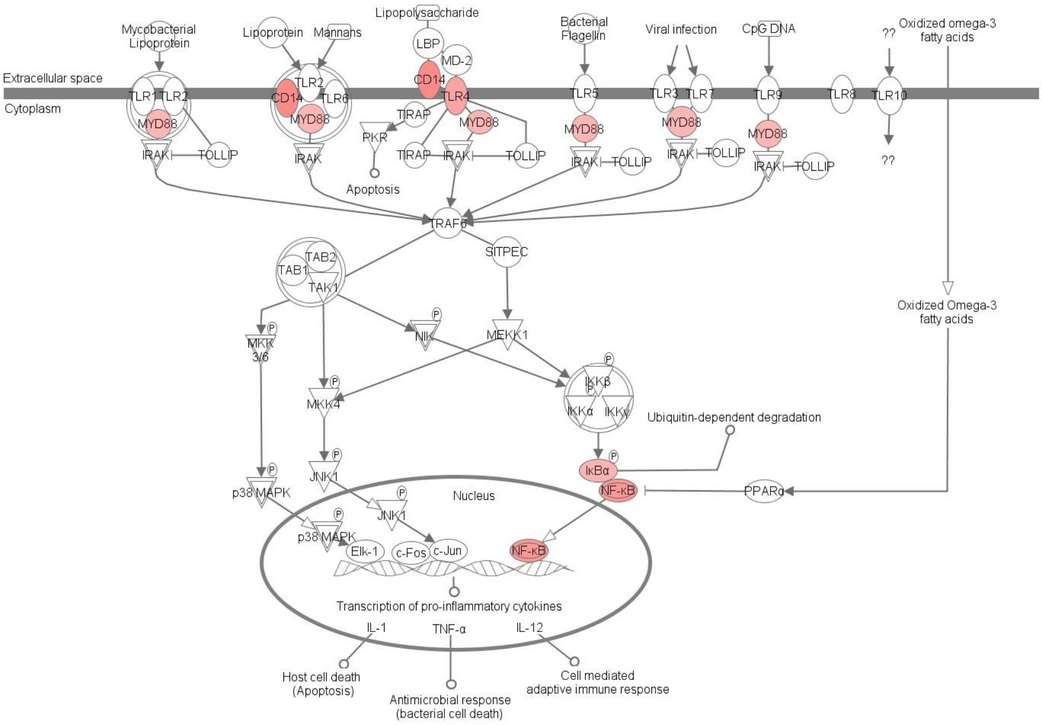

© 2000-2011 Ingenuity Systems, Inc. All rights reserved.

Acute Phase Response Signaling

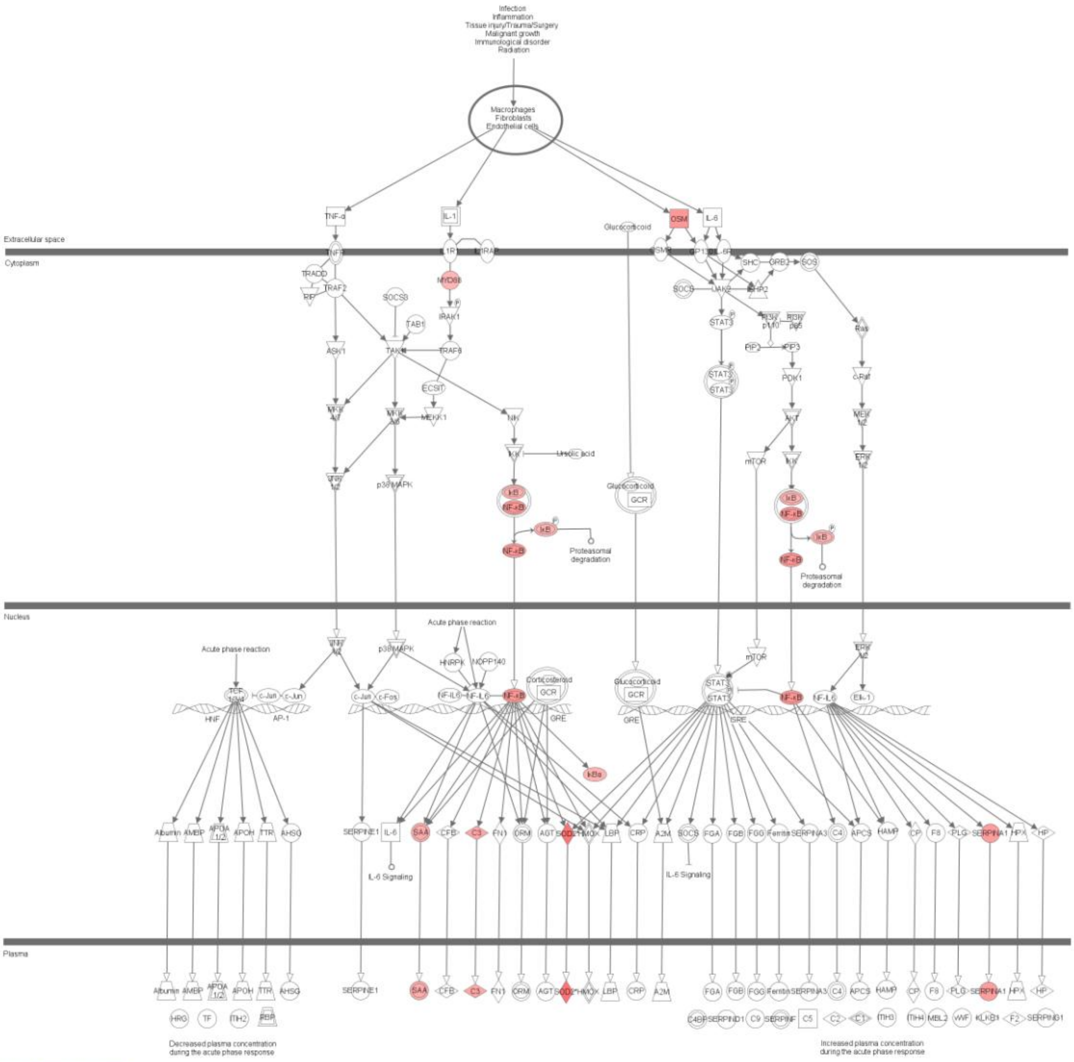

© 2000-2011 Ingenuity Systems, Inc. All rights reserved.

MIF-mediated Glucocorticoid Regulation

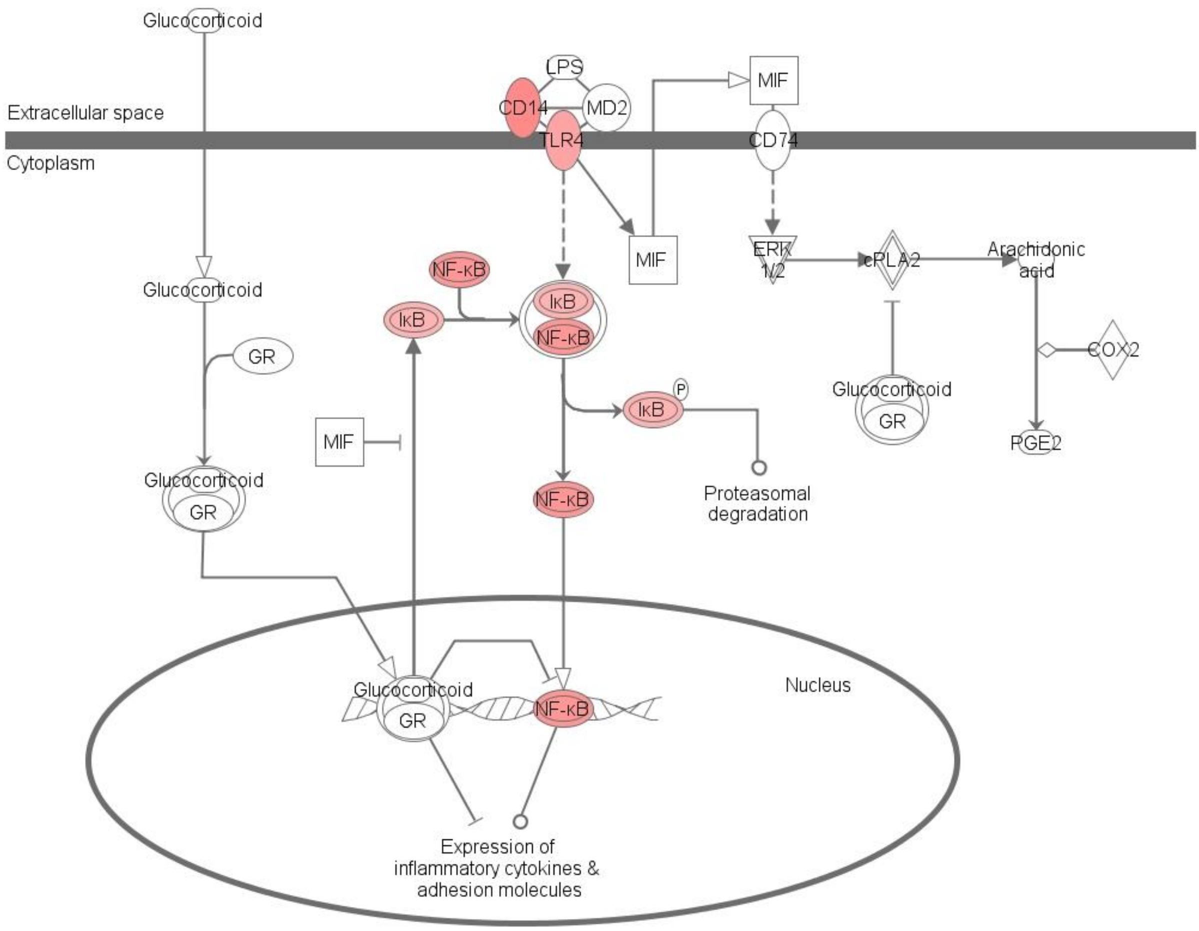

© 2000-2011 Ingenuity Systems, Inc. All rights reserved.
