# Supplementary figures and images for: Response of the goat mammary gland to infection with Staphylococcus aureus revealed by gene expression profiling in milk somatic and white blood cells
Source: BMC Genomics. 2012 Oct 9;13:540. doi: 10.1186/1471-2164-13-540 (PMC3532242; doi:10.1186/1471-2164-13-540)

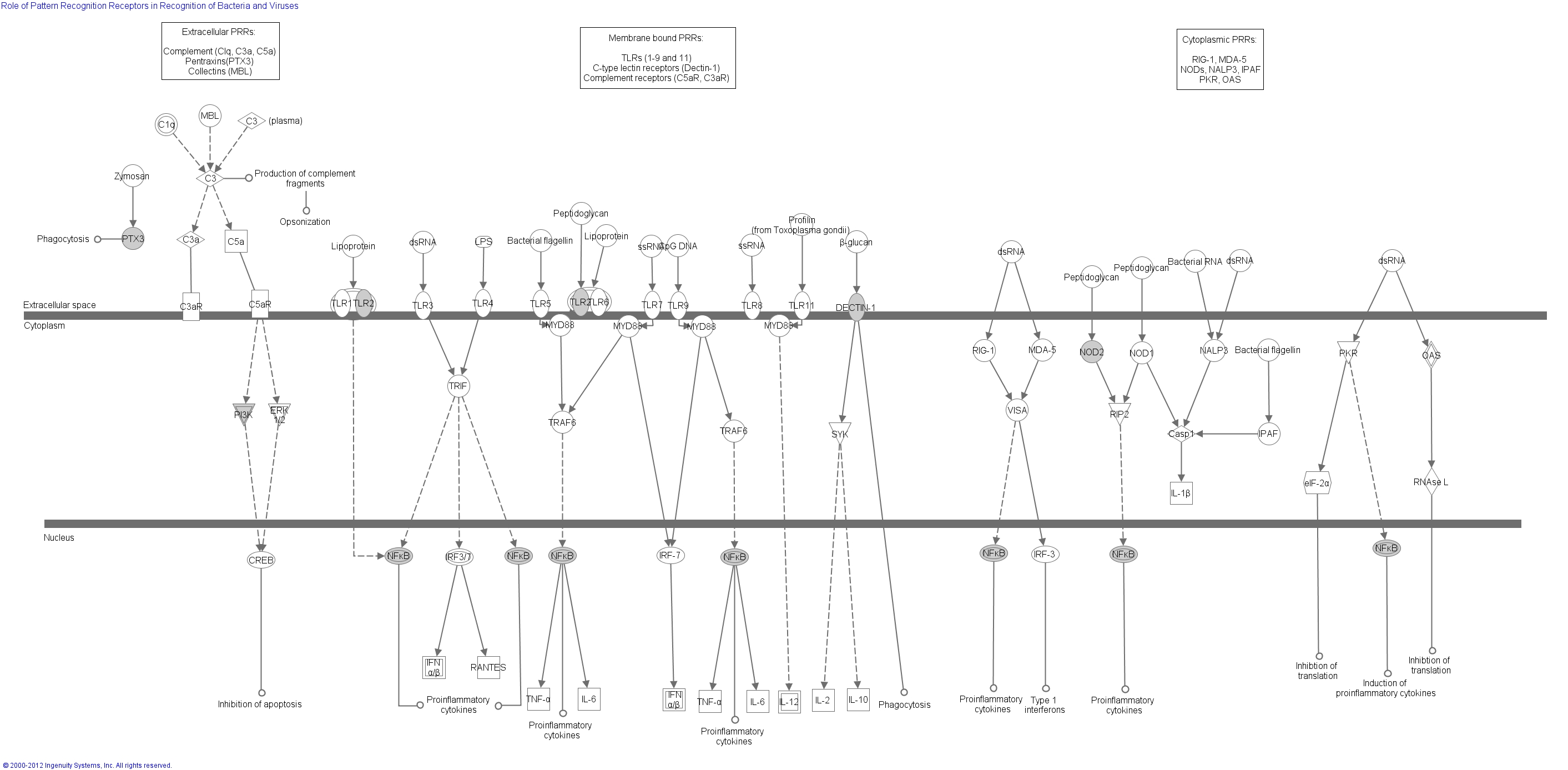

Supplement: Additional file 5 — Role of pattern recognition receptors in recognition of bacteria and viruses pathway. Canonical pathway performed with IPA Knowledge Base. [file 1471-2164-13-540-S5.jpeg]
